# Deep learning for the classification of atrial fibrillation using wavelet transform-based visual images

**DOI:** 10.1186/s12911-025-02872-5

**Published:** 2025-01-21

**Authors:** Ling-Chun Sun, Chia-Chiang Lee, Hung-Yen Ke, Chih-Yuan Wei, Ke-Feng Lin, Shih-Sung Lin, Hsin Hsiu, Ping-Nan Chen

**Affiliations:** 1 School of Medicine, National Defense Medical Center, Taipei, 11490 Taiwan; 2 Graduate Institute of Applied Science and Technology, National Taiwan University of Science and Technology, Taipei, 10607 Taiwan; 3 Division of Cardiovascular Surgery, Department of Surgery, Tri-Service General Hospital, National Defense Medical Center, Taipei, 11490 Taiwan; 4 Graduate Institute of Life Sciences, National Defense Medical Center, Taipei, 11490 Taiwan; 5 School of Public Health, National Defense Medical Center, Taipei, 11490 Taiwan; 6 Medical Informatics Office, Tri-Service General Hospital, National Defense Medical Center, Taipei, 11490 Taiwan; 7 Department of Computer Science and Information Engineering, Chinese Culture University, Taipei, 11114 Taiwan; 8 Graduate Institute of Biomedical Engineering, National Taiwan University of Science and Technology, 10607 Taipei, Taiwan; 9 Department of Biomedical Engineering, National Defense Medical Center, Taiwan, No.161, Sec.6, Minchiuan E. Rd., Neihu Dist, Taipei, 11490 Taiwan

**Keywords:** Atrial fibrillation, MsCWT, Convolutional Neural Network, ResNet101

## Abstract

**Background:**

As the incidence and prevalence of Atrial Fibrillation (AF) proliferate worldwide, the condition has become the epicenter of a plethora of ECG diagnostic research. In recent diagnostic methodologies, Morse Continuous Wavelet Transform (MsCWT) is a feature extraction technique utilized to draw out distinctive attributes of ECG signals. In our study, we explore the employment of MsCWT in the classification of AF with ECG signals in a continuum.

**Results:**

We present a MsCWT image-based deep learning machine for AF differentiation. For the training, validation, and test sets, we achieved average accuracies of 97.94%, 97.84%, and 91.32%; and overall F1 scores of 97.13%, 96.86%, and 89.41% respectively. Moreover, AUC ROC curves of over 0.99 were obtained for all classes in the training and validation sets; and were over 0.9679 for the test set.

**Conclusions:**

Training deep learning machines for the classification of AF with MsCWT-based images demonstrated to yield favorable outcomes and achieved superior performance amongst studies utilizing the same dataset. Though minimal, the conversion of signals into wavelet form with MsCWT may drastically improve outcomes not only in future ECG signal studies; but all signal-based diagnostics.

## Background

Atrial Fibrillation (AF) occurs when there is electrical disarray in the heart, resulting in an irregular heartbeat. The condition is associated with a multitude of adverse health events and complications, including myocardial infarctions [1], severe acute ischemic stroke [2], and dementia [3]. Early treatment of the condition prompts favorable cardiovascular outcomes [4]; thereby, early diagnosis—to initiate treatment—is essential to facilitate optimal patient prognosis. At the writing of this literature, there are over 37,574 million individuals affected by AF worldwide [5]; and as the incidence and prevalence of the condition are forecasted to climb for the next few decades [5], AF diagnosis becomes increasingly pertinent and crucial in the present day.

In clinical practice, AF diagnosis necessitates a time-consuming manual electrocardiogram (ECG) interpretation by physicians [6–8]. Though a considerable amount of time is expended, a meta-analytic study on the accuracy of physicians’ ECG interpretations demonstrated the average diagnostic accuracies by physicians are only just over 50 percent [9]. To safeguard patients from potentially harmful medical treatments due to misdiagnosis [10] and to elevate physicians’ diagnostic accuracy, researchers have studied and developed artificial intelligence (AI)-based methodologies to detect AF [11–21].

Recently, Klosowski [11] compared training with raw and spectral-extracted ECG signals in the classification of differential cardiac dysfunctions—including AF—and proved that training with spectral-extracted ECG signals has an edge over raw ECG signals. The study reported a classification accuracy of 100 percent for their test set, however, the accuracy achieved is not reliable as their test set only contains 60 images for each class. To further evaluate the viability of feature extraction with spectral entropy, the concept was implemented by Fang [12] in the detection of AF with dual-channel neural networks and by Lee [13] in the classification of arrhythmias—including AF—with a convolutional neural network (CNN). In both studies, classification accuracies of AF were only just over 80 percent. Though the technique is relatively new and spectrally extracted ECG signals does exhibit an advantage over raw ECG signals in the detection of AF; it does not push boundaries in AF detection.

AF classification approaches employing wavelets: stationary wavelet transforms [14, 15], discrete wavelet transforms [16, 17], and continuous wavelet transforms [18, 19], have previously been demonstrated to reach accuracies of over 90 percent; and due to wavelets’ capacity to foster robust and accurate classification machines, continued variants of the technique are still explored.

While probing into wavelet transform variants, Wachowiak [20] proposed the application of Morse Continuous Wavelet Transform (MsCWT) to analyze electromyography (EMG) and ECG signals of skaters; and substantiated the feasibility of this method to explore undetected frequency features in the time domain. This approach was further delved into by Wang [21] and was able to attain an accuracy of 98.74 percent on the MIT-BIH arrhythmia database. Wang’s application of using an MsCWT approach to analyze ECG signals consisted of translating individual beats (PQRST waves) in ECG data into wavelet form and utilizing the wavelet form beats alongside R-R interval information to construct a CNN-based classification machine. However, despite achieving a high classification accuracy, the aforementioned machine performed underwhelmingly in terms of sensitivity at 67.47 percent; meaning, a large proportion—over 30 percent—of diseased patients were falsely diagnosed as not diseased. This outcome may be due to the limited scope of the machine, as it was built using only individualized beats and R-R interval information; and would be incapable of perceiving the dynamic and subtle features of ECG signals in a continuum.

Thus, in this study, we explore the application of Morse Continuous Wavelet Transform in the quantification of 30 s single-lead ECG signals. For findings to be comparative with related works, the publicly available arrhythmia dataset from the PhysioNet/Computing in Cardiology Challenge 2017 was utilized to build and evaluate the performance of our approach [22]. The outcomes of our study paves way for future diagnostic approaches utilizing MsCWT to analyze data in a continuum; and further, validate the prospect of machines to accelerate physicians’ tasks and rectify potential errors in physician judgment.

## Methods

### System overview

**Fig. 1:**
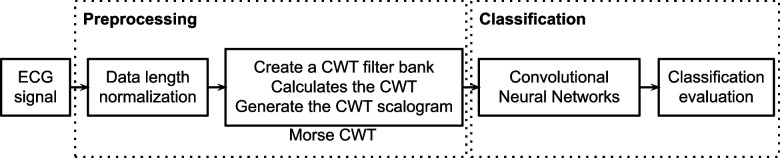
Flowchart of our proposed method

In this study, we used the dataset from the PhysioNet/Computing in Cardiology Challenge 2017 to evaluate the proposed method. Our method consists of two stages: preprocessing and classification. The preprocessing stage incorporates data length normalization and morse continuous wavelet transformation; and the classification stage involves convolutional neural networks and classification evaluation. A more comprehensive outline of our methodology is described in further sections. The flowchart below Fig. 1 summarizes our proposed method.

### Dataset and ECG preprocessing

We trained a neural network to classify cardiac arrhythmias using the dataset from the PhysioNet/Computing in Cardiology Challenge 2017. The dataset contains four types of single-lead ECG recordings (8,528) of which the sampling rate was 300 Hz for recordings of 9 to 60 s (2,700 to 18,000 bytes). ECG recordings are segmented (preprocessing) with the algorithm below:

**Figure:**
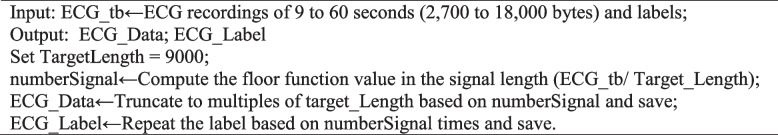
**Algorithm 1.** Segmentation the ECG Recordings

**Fig. 2:**
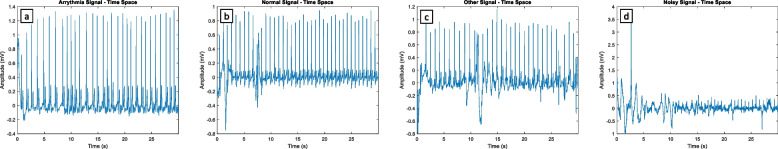
ECG Waveforms of (**a**) normal rhythm, (**b**) AF rhythm, (**c**) other rhythm and (**d**) noisy recordings

In essence, for preprocessing, 30 s cuts from the ECG samples were selected for experimentation; and samples which fell short of 30 s were excluded from our study. Subsequently, classes: atrial fibrillation (AF), normal rhythm (Normal), other rhythms (Other), and noisy (Noisy) consisted of 718, 4937, 2603, and 151 images respectively; thus, the final data count used for this study decreased from 8,528 to 8,409. The full breakdown for each class and each subset is reported below in Table 1. ECG waveforms of (a) normal rhythm, (b) AF rhythm, (c) other rhythm and (d) noisy recordings are shown in Fig. 2a-d.

**Table 1:** Breakdown of Training, Validation, and Test Sets for the PhysioNet/Computing in Cardiology 2017 Dataset

Condition	PhysioNet 2017 Dataset			
	Training (64%)	Validation (16%)	Test (20%)	Total (100%)
AF	459	115	144	718
Normal	3,160	790	987	4,937
Other	1,666	416	521	2,603
Noisy	97	24	30	151
Total	5,382	1,345	1,682	8,409

### Short-Time Fourier Transform (STFT)

The four classes of ECG signals are composed of different frequency components, thereby time–frequency analysis of the signals result in differential signal resolutions. STFT is used to analyze how the frequency of a non-stationary signal changes over time. To distinguish the differences between ECG signals, we use a short-time Fourier transform technique to decompose the ECG signals into features of time and frequency vectors. A STFT on a time-domain signal *x*(*t*) generates a complex-valued matrix X, where $\omega (\tau )$ is the Hamming window function; moreover, the STFT of a signal *x*(*t*), can be defined as:

1 $$STFT\left\{x\left(t\right)\right\}=X(\tau , \omega )=\underset{-\infty }{\overset{\infty }{\int }}x(t)\omega (t-\tau ){e}^{-j\omega t}dt$$

**Fig. 3:**
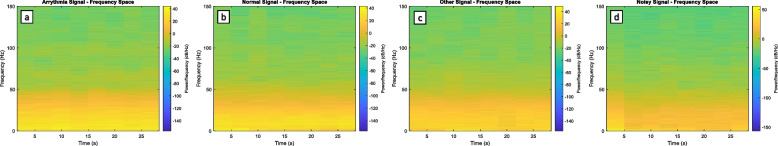
ECG signal represented with the STFT in the time- frequency domain. (**a)** normal rhythm, (**b**) AF rhythm, (**c**) other rhythm and (**d**) noisy recordings

ECG signal with sampling rate of 128 Hz–denoted by $x\left(t\right)$ –and a 256-point Hamming window length with 128-point overlap was utilized to produce STFT transformations; and are shown in Fig. 3a-d. The two-dimensional visualization is on two coordinates, one related to the window time frame (τ) and the other to the frequency (ω). However, though the resulting spectrograms seemed to have demonstrated differences between signals, the extent was limited and lacked pronounced distinctions, and thus was ultimately not employed in our study.

### Continuous Wavelet Transform (CWT) and scalogram representations

**Fig. 4:**
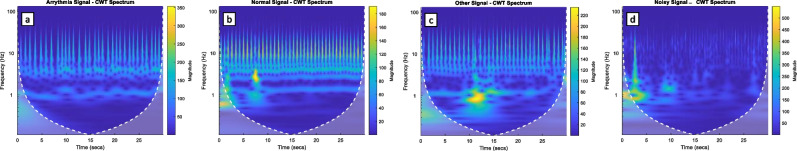
ECG signal represented with the CWT scalogram. (**a)** normal rhythm, (**b**) AF rhythm, (**c**) other rhythm and (**d**) noisy recordings

In this study, we used the continuous wavelet function to decompose the signal to obtain a clear time–frequency representation of the signal. Wavelet transform of a signal *x*(*t*) is defined as

2 $$Z\left(a, b\right)=\frac{1}{\sqrt{a}}\underset{-\infty }{\overset{\infty }{\int }}x(t){\psi }^{*}\frac{(t-b)}{a}dt$$

where *a* is a scale parameter, *b* is a translation parameter, and $\psi (t)$ is the wavelet function. A scalogram is the absolute value of the CWT coefficients of a signal. First, we built a continuous wavelet transform filter bank using Morse (3, 60) wavelets; then, with continuous wavelet, the 30-s ECG signals were used to calculate wavelet coefficients and frequencies. The continuous wavelet calculation was done by utilizing wavelets of different scales to execute a window-by-window convolution operation to obtain the wavelet coefficient matrix. Finally, by combining the wavelet coefficients of different frequencies, a time–frequency transformed wavelet coefficient map was obtained. Figure 4a-d provides a comparison between the four classes of ECG.

**Fig. 5:**
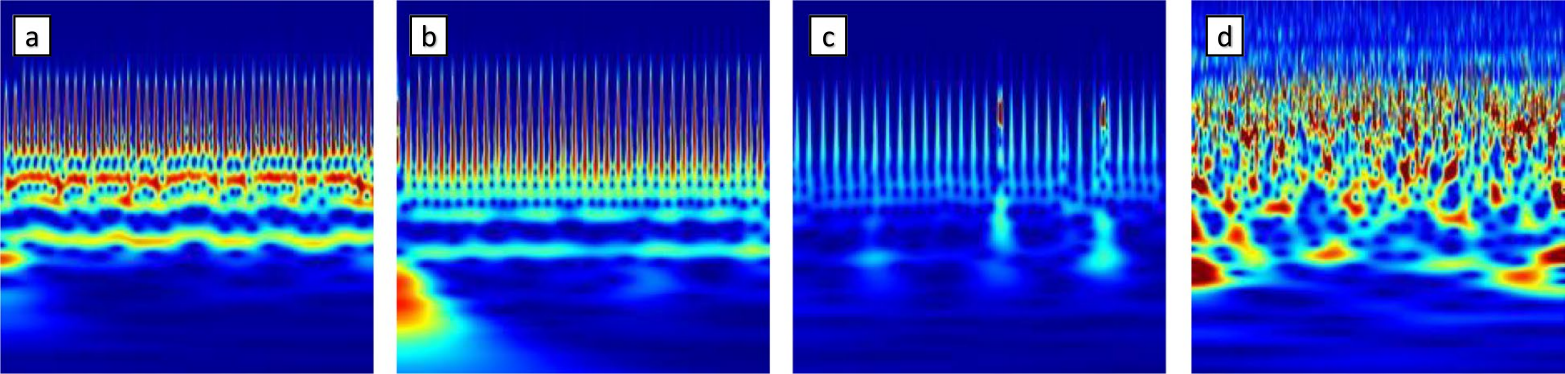
Energy scalograms of ECG signals. **a** AF rhythm (no. A_3111.jpg); **b** normal sinus rhythm (no. N_181.jpg); **c** Other rhythm (no. O_119.jpg); **d** Noisy (no. ~ _7909.jpg)

The energy scalograms for four categories (atrial fibrillation, normal, other, and noise) are shown in Fig. 5 to illustrate input data for deep learning training. In Fig. 5(a), the AF rhythm (no. A_3111.jpg) is depicted, while Fig. 5(b) shows a normal sinus rhythm (no. N_181.jpg). It's evident from the figures that the peaks of each signal differ, with areas of high energy concentration appearing yellow. This color distribution enables us to differentiate between arrhythmia and normal signals. Although some activities are visible in the lower half of the atrial fibrillation ECG scalogram, none are present in the normal rhythm ECG scalogram. Energy scalograms effectively highlight distinctive features of ECG signals, demonstrating the potential of this approach in elucidating different ECG signal features to improve the performance of deep learning models.

### Convolution Neural Network (CNN)—based ECG Classification

In our study, transfer learning from a pretrained CNN was implemented for the classification of AF, Normal, Other, and Noisy. The pretrained CNN: ResNet101, selected from Matlab's 2022a Pretrained CNN document [23], was employed for our research.

The pre-trained ResNet101 network is comprised of five convolutional (Conv) blocks and a final fully connected (FC) layer. The first convolution block (Conv1) consists of a single layer and has a 7 × 7x64 filter. Following the first block, there is a 3 × 3 max pooling layer which is subsequently succeeded by 4 blocks, each block with 3 convolution layers.

**Fig. 6:**
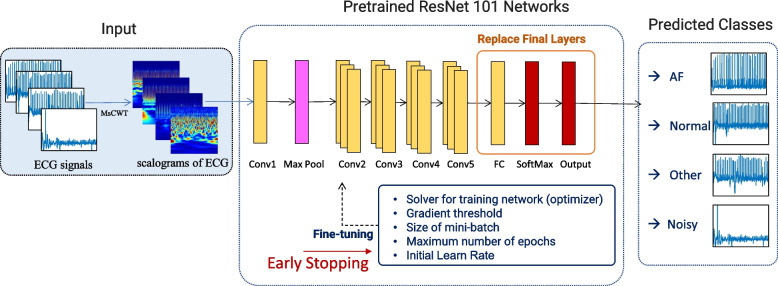
The architecture of ECG Classification Based on ResNet 101 Networks

The CNN architecture for this study is shown in Fig. 6**.** We replaced the last 3 layers (fully connected layer, softmax layer, and classification layer) of the pre-trained model with a new layer and trained with only the new layers. And ECG scalograms with an array of size 224 × 224x3 RGB images were used as input into the convolutional network architecture.

To fine-tune the pretrained network, we tune the following hyperparameter values: solver for training network (optimizer), gradient threshold, size of mini-batch, maximum number of epochs, and initial learn rate. In addition, we also used an early stopping method, which stopped training when model performance on the validation dataset stops improving. When there is no improvement in validation accuracy (validation lag) after 10 iterations, the early stopping mode of the training process is triggered and stops further training.

Our deep learning machine was trained using the root mean square propagation (rmsprop) optimization algorithm with a learning rate of 0.0001, a mini-batch size of 64, and a maximum epoch of 105. Before training, the image resolution was normalized to meet the corresponding pre-trained CNN input requirements. As stated previously, at this stage, the dataset was randomly split into training, validation, and testing in a ratio of 64:16:20.

### Classification evaluation

The performance of the algorithms was evaluated on their capacity to accurately classify images of the training, validation and test sets based on: accuracy (Acc), sensitivity (Se), specificity (Sp), Precision (Pre), F1 score (F1) and Matthew’s correlation coefficient (MCC). The inclusion of MCC further elevates the reliability of evaluation overall [24]. The six statistical metrics are defined in the following equations: (in which Tp: true positive, Fn: false negative, Tn: true negative, Fp: false positive)

$$\text{Accuracy }(\text{Acc})=\frac{{\varvec{T}}{\varvec{p}}+{\varvec{T}}{\varvec{n}}}{{\varvec{T}}{\varvec{p}}+{\varvec{T}}{\varvec{n}}+{\varvec{F}}{\varvec{p}}+{\varvec{F}}{\varvec{n}}}$$

$$\text{Sensitivity }(\text{Se})=\frac{{\varvec{T}}{\varvec{p}}}{{\varvec{T}}{\varvec{p}}+{\varvec{F}}{\varvec{n}}}$$

$$\text{Specificity }(\text{Sp})=\frac{{\varvec{T}}{\varvec{n}}}{{\varvec{T}}{\varvec{n}}+{\varvec{F}}{\varvec{p}}}$$

$$\text{Precision }(\text{Pre})=\frac{{\varvec{T}}{\varvec{p}}}{{\varvec{T}}{\varvec{p}}+{\varvec{F}}{\varvec{p}}}$$

$$\text{F}1-\text{score }(\text{F}1)=\frac{2\times \text{Sensitivity}\times \text{Precision}}{\text{Sensitivity}+\text{Precision}}$$

$$\text{Matthews correlation coefficient }(\text{MCC})=\frac{{\varvec{T}}{\varvec{p}}\times {\varvec{T}}{\varvec{n}}-{\varvec{F}}{\varvec{p}}\times {\varvec{F}}{\varvec{n}}}{\sqrt{\left({\varvec{T}}{\varvec{p}}+{\varvec{F}}{\varvec{p}}\right)\left({\varvec{T}}{\varvec{p}}+{\varvec{F}}{\varvec{n}}\right)\left({\varvec{T}}{\varvec{n}}+{\varvec{F}}{\varvec{p}}\right)\left({\varvec{T}}{\varvec{n}}+{\varvec{F}}{\varvec{n}}\right)}}$$

## Results

The analysis and model development of the proposed algorithms was performed on a workstation with the following hardware configuration and specifications: Intel® Xeon® Platinum 8165 Processors, RAM 128 GB, 3 GPUs NVIDIA® Tesla® P100 16 GB memory. The experiments ran on Matlab 2022a Deep Learning Toolbox in the 64-bit Windows 10 operating system.

**Fig. 7:**
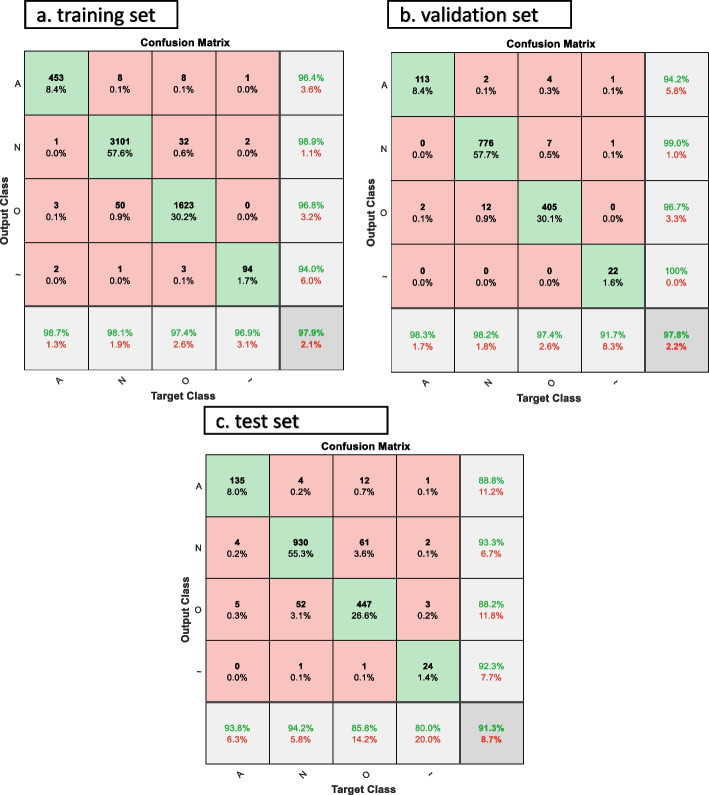
Confusion matrix for atrial fibrillation (A), normal rhythm (N), other rhythms (O), and noisy ( ~) four classes identification based on MsCWT and residual neural network. Confusion matrix for (**a**) training set, (**b**) validation set, and (**c**) test set

The AF classification results using MsCWT and CNN designed are shown in Table 2. Performance was evaluated using metrics: accuracy, sensitivity, specificity, precision, F1 score, and MCC score. We achieve high performance in all classes in the training, validation, and test sets with average accuracies of 97.94%, 97.84%, and 91.32%; and overall F1 scores of 97.13%, 96.86%, and 89.41% respectively. Figure 7 shows the confusion matrix for the MsCWT and residual neural network-based AF classification method. The diagonal elements represent the number of points where the predicted label is equal to the true label. The red squares show the number of misclassifications for each class.

**Table 2:** AF classification performance on train, validation, and test datasets using ResNet101 models

Classifiers	Acc (%)			Sen (%)	Sp (%)		Pre (%)			F1 (%)		MCC (%)
Training Set (64%, 5382 images)												
AF	98.69			98.69	99.65		96.38			97.52		97.30
Normal	98.13			98.13	98.42		98.88			98.51		96.41
Other	97.42			97.42	98.57		96.84			97.13		95.83
Noisy	96.91			96.91	99.89		94.00			95.43		95.36
Average	97.94			97.79	99.13		96.53			97.15		96.22
Validation Set (16%, 1345 images)												
AF	98.26			98.26	99.43		99.84			96.17		95.83
Normal	98.23			98.23	98.56		97.50			98.60		96.64
Other	97.36			97.36	98.49		98.81			97.01		95.66
Noisy	91.67			91.67	100.00		99.85			95.65		95.67
Average	97.84			96.38	99.12		99.00			96.86		95.95
Test Set (20%, 1,682 images)												
AF	93.75			93.75	98.89		88.82			91.22		90.41
Normal	94.22			94.22	90.36		93.28			93.75		84.77
Other	85.80			85.80	94.83		88.17			86.96		81.25
Noisy	80.00			80.00	99.88		92.31			85.71		85.70
Average	91.32			88.44	95.99		90.64			89.41		85.53

*Acc* Accuracy, *Sen* Sensitivity, *Sp* Specificity, *Pre* Precision, *F1* F1 score, *MCC* Matthew’s correlation coefficient

**Fig. 8:**
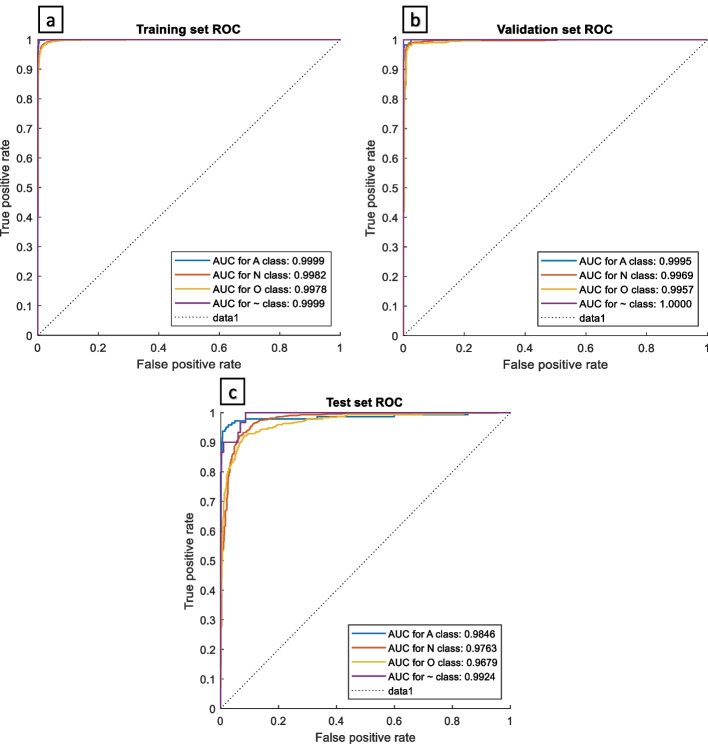
Receiver Operating Characteristic Curve for every class. **a** Using training set, **b** validation set, and **c** test set Dataset. The dotted line (data1) shows a worthless test that has a 50/50 chance of correctly detecting it

The receiver operating characteristic (ROC) curves and the area under the curve (AUC) values of the four ECG signal waveforms in the training set, validation set and test set: AF, normal, other and noise groups are shown in Fig. 8**.** We obtained over 0.99 AUC ROC curves for all classes in training and validation sets. On the test set, all classes obtained AUC ROC curves of more than 0.9679. Notably, our method achieved AUC ROC curves of more than 0.98 for atrial fibrillation in all sets.

**Fig. 9:**
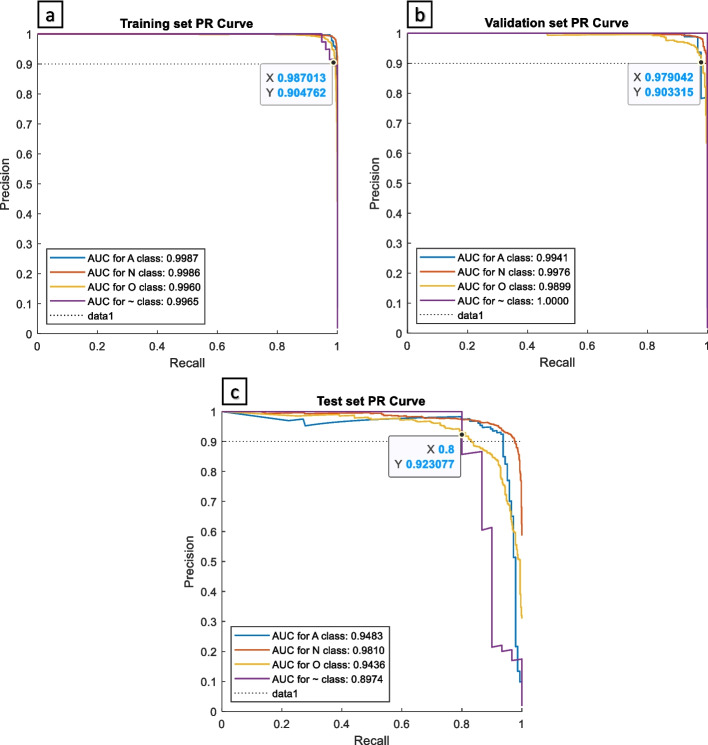
Precision-recall curves for every class. **a** Using training set, (**b)** validation set, and (**c**) test set Dataset. The dotted line (data1) indicates precision is 0.9

We use the precision-recall curve (PR curve) to analyze the effectiveness of the proposed model; as in evaluation of the efficacy of machines with unbalanced input classes, the PR curve is superior to the receiver operating characteristic curve (ROC curve) [25]. As shown in Fig. 9, we obtained over 0.98 in AUC PR curves in the training and validation sets for all classes. On the test set, all classes obtained AUC PR curves of more than 0.8974. We obtained a high-yielding AUC PR curves of more than 0.94 for atrial fibrillation in all sets. If the precision is set at 0.9; the lowest recall of the training set (noise) is 0.987; the lowest recall of the validation set (other rhythms) is 0.978; and the lowest recall of the test set (noise) is 0.8. Thereby, even when precision is set at 0.9; our machine can still reach recall scores of 0.8 up to 0.987. Overall, even with unbalanced input classes, our proposed MsCWT and CNN method demonstrates to be effective.

## Discussion

Our wavelet-based deep learning approach not only rectifies the previously addressed poor sensitivity issue, but also achieved high accuracies with each set; and with no performance metric below 80.00 percent, demonstrated an overall sound performance. The 30 s single-lead ECG signals for training quantifies a holistic view of AF; thus, presenting a more physiological and pragmatic diagnostic approach to the condition: instead of looking for ‘particular ECG components,’ the approach surveys for features based on the physiological progression of AF presented in the 30 s ECG recording. As clinical scenarios are complex, patients may not exhibit traditional AF – like components in the ECG, thus, an approach that follows the physiological mechanism is advantageous over one that only inspects for components; and thereby more pragmatic.

Several studies on the classification of ECG data have been previously conducted employing the same dataset from the PhysioNet/Computing in Cardiology Challenge 2017 database. Table 3 shows the comparison of our proposed method (in bold) with other methods in terms of the test set. Xiong [26] and Hsieh [27] both used 1D-CNN for feature extraction, and achieved F1 scores of 80.8% and 85.8% on AF, respectively. Jiang [28] adopted the HADLN architecture, and combined it with ResNet and Bi-LSTM 1D-CNN modules, to effectively solve the problem of gradient dispersion when increasing the number of network layers; and obtained a F1 score of 88%, which is the highest accuracy of all 1D CNN studies. Zhao [29] proposed an efficient 2D deep convolutional neural network to classify 2D spectral-temporal ECG data; and achieved a F1 score of 79.18% on AF. Fang [12] used Typical spectrograms and Poincare plot techniques to convert ECG signals into different 2D images respectively and used VGG16 network for training; and obtained a final F1 score of 89% on AF. Lee [13] use the ECM method to convert each beat into a 2D image and used the BIT-CNN model to implement AF training and classification, and their final F1 score on AF was 89.73%. As demonstrated in the aforementioned articles, in comparison with 1D-CNN, 2D-CNNs methodologies achieved higher accuracies in the classification of heart rhythm. In this study, we use the 2D-CNN method combined with MsCWT and ResNet101 CNN; and our proposed method obtained F1 values of 91.22, 93.75, 86.96, 85.71 for AF, Normal, Other, Noisy respectively.

**Table 3:** Comparison of our work with related CNN-based methods with the test set

Study	Classifiers	F1(AF)	F1(Normal)	F1(Other)	F1(Noisy)
Hsieh [27]	1D CNN	80.80	90.40	66.20	75.30
Xiong [26]	1D CNN	85.80	91.90	81.60	-
Jiang [28]	1D ResNet + Bi-LSTM	88.00 (weight average)			
Zhao [29]	2D Kalman CNN	79.18	89.29	72.25	52.50
Fang [12]	2D Dual-Vgg16	83.00	90.00	75.00	83.00
Lee [13]	2D ECM Bit-CNN	89.73	81.06	74.45	62.22
**Our work**	**2D MsCWT CNN**	**91.22**	**93.75**	**86.96**	**85.71**

## Conclusions

Our deep learning approach based on MsCWT and ResNet101 CNN achieved favorable outcomes; and also rectified the previously addressed poor sensitivity of a past study. By building a machine based on 30 s time frames of AF, the progression of ECG changes is also recorded and employed within training; thereby, rather than scrutinizing for classical ECG components of AF, our machine detects AF-caused ECG changes – which sometimes may not be inclusive of classical AF features. Moreover, not only does our study substantiate the plausibility and validity of utilizing AI to support doctors in detecting AF; it also sets a benchmark for future MsCWT based experiments utilizing ECG signals in a continuum.

Our findings can be of a useful reference for the development of wearable medical devices capable of reducing the risk of autonomic dysregulation during physical training by alerting wearers of AF onset. However, rather than detecting the onset of AF; predicting AF onset could also greatly minimize potential health complications. Thereby, to identify distinct patterns and sequences which could prospectively forecast the onset of AF; a future study could utilize 24—48-h or perhaps even longer ECG recordings of AF patients. In addition, physicians could work with patients to deduce and resolve particular habits and or activities that precipitate certain patterns and sequences.

## Data Availability

Publicly available datasets were analyzed in this study. This data can be found here: https://physionet.org/content/challenge-2017/1.0.0/

## Abbreviations

AF: Atrial fibrillation
MsCWT: Morse Continuous Wavelet Transform
ECG: Electrocardiogram
CWT: Continuous Wavelet Transform
CNN: Convolutional neural network
AUC: Area under the receiver operating characteristic curve
ROC: Respective receiver operating characteristic curve
AI: Artificial intelligence
EMG: Electromyography
STFT: Short-Time Fourier Transform
RGB: Red, green, blue
Conv: Convolutional
FC: Fully connected
Conv1: The first convolution block
rmsprop: Root mean square propagation
Acc: Accuracy
Se: Sensitivity
Sp: Specificity
Pre: Precision
F1: F1 score
MCC: Matthews correlation coefficient
Tp: True positive
Fn: False negative
Tn: True negative
Fp: False positive
PR curve: Precision-recall curve
AUC PR curves: Area under the precision-recall curve
LSTM: Long Short-Term Memory
